# Editorial: Novel approaches in the prevention of bacterial biofilm formation

**DOI:** 10.3389/fcimb.2023.1212386

**Published:** 2023-07-07

**Authors:** Shalin Parikh, Jayendrakumar Patel, Rakesh Patel, Ravi Patel

**Affiliations:** ^1^ Shree SK Patel College of Pharmaceutical Education and Research, Ganpat University, Mehsana, Gujarat, India; ^2^ Pharmaceutical Science, Exemplify Biopharma Inc., Cranbury, NJ, United States; ^3^ Graduate School of Pharmacy, Gujarat Technological University, Gandhinagar, Gujarat, India

**Keywords:** biofilm, biofilm formation, *Pseudomonas aeruginosa*, poly lactic-co-glycolic acid (PLGA), catheters, nanoparticles, daphnetin

This editorial provides an outline of the papers published in the Frontiers Research Topic “*Novel approaches in the prevention of bacterial biofilm formation*” in the journal Frontiers in Cellular and Infection Microbiology. Biofilms are cultures of bacteria that have organized themselves inside a polymer matrix made up generally of polysaccharides, proteins, and extracellular DNA that the bacteria manufacture entirely or partially (Vestby et al., 2020).Nature has endowed bacteria with the capacity to build biofilms, which severely threaten humankind. Bacterial biofilms are impervious to the body’s innate and adaptive inflammatory defense mechanisms, including antibiotics and disinfection chemicals, as well as phagocytosis. Preventing the development of bacterial biofilms is a promising strategy for lowering the prevalence of pathogenic microorganisms, and emerging techniques for accomplishing this goal require immediate attention (Hall-Stoodley et al., 2004).Through both fundamental and practical research, the Research Topic offers a view on the novel approaches in the prevention of bacterial biofilm formation. In addition, the articles examine a multi-faceted approach to preventing bacterial biofilms using a new chemical substance and exploring new formulation technologies (Iolanda and Gianfranco, 2010; Chen et al., 2013).The goal is to create a commercial solution for patients who require it. This editorial comprises seven publications that focus on the current research related to the prevention of the formation of bacterial biofilms along with a critical review of it. In a study titled “Silicone Foley catheters impregnated with microbial indole derivatives inhibit crystalline biofilm formation by *Proteus mirabilis*”, Amer et al. sought the use of microbial indole extract in catheters can effectively decrease the formation of crystalline biofilm caused by *P. mirabilis*. Acting on virulence factors at sub-MIC levels without stressing bacterial growth lowers resistance development. This chemical is also safe for human cell lines. This indole extract reduces crystalline biofilm formation by inhibiting virulence factors that affect bacterial motility, adherence, and urease production. Long-term catheterization in animal models and synergistic action with additional anti-virulence drugs are planned to test this technique. The following publication, titled “Effects of Daphnetin on Biofilm Formation and Motility of *Pseudomonas aeruginosa*”, aims to evaluate the effects of daphnetin on biofilm formation and motility of *Pseudomonas aeruginosa*. Antimicrobial effects against *P. aeruginosa* were demonstrated by Daphnetin at high concentrations, while anti-biofilm effects were observed at concentrations that did not hinder bacterial growth. Daphnetin has the potential to serve as a new antibacterial and anti-biofilm agent against *P. aeruginosa*. Further research is necessary to clarify the pharmacokinetic and pharmacodynamic characteristics of Daphnetin in animals infected with *P. aeruginosa* biofilms prior to its clinical trial initiation. The results of this study offer some cause for optimism about the possible application of daphnetin in the management of the production of bacterial biofilms. The Research Topic also discovers the promising usage of nanoparticles in biology in general and in microbiology particularly as Bottagisio et al. have illustrated in both the following experiments. The study titled “Vancomycin-nano functionalized peptide-enriched silk fibroin to prevent methicillin-resistant *Staphylococcus* epidermidis-induced femoral non-union in rats” from Bottagisio et al. investigated combining nanotechnology for local antibiotic delivery with the osteoconductive properties of peptide-enriched silk fibroin antibiotic functionalized nanoparticles (AFN-PSF) sponges could potentially prevent infection and stimulate bone growth in the presence of infected fractures synthesized with metal plates. It was discovered that AFN-PSF has enough antimicrobial activity to prevent bone infection *in vivo*, as demonstrated in a rat femoral fracture model where a methicillin-resistant *S. epidermidis* (MRSE) strain was inoculated. The study titled “Exploring multielement nanogranular coatings to forestall implant-related infections” by Bottagisio et al. concludes that Ti-Ag-Cu and Mg-Ag-Cu coatings have effective anti-adhesive properties and can prevent biofilm formation. The study suggests that these multi-element nanoparticles can be used safely as a new strategy to prevent bacterial attachment to biomedical implant surfaces. Different behavior was noticed in the culture of *Pseudomonas aeruginosa* on Ti-Ag-Cu and Mg-Ag-Cu-coated discs after 30 and 120 minutes. The active effect of Mg-Ag-Cu was found to mainly reduce biofilm formation compared to Ti-Ag-Cu. Coatings had a milder effect on *P. aeruginosa*, which is likely due to its exceptional capability of attachment and matrix production. The evaluation of bacterial colonization on nanoparticle-coated discs through confocal microscopy confirmed the data. The review titled “PLGA-Based Nanoplatforms in Drug Delivery for Inhibition and Destruction of Microbial Biofilm” by Shariati et al., poly lactic-co-glycolic acid (PLGA) has the potential to serve as a delivery system for a range of anti-biofilm agents, including antibiotics, natural compounds, and enzymes. However, there is a lack of established standards and guidelines for evaluating the effectiveness, safety, and performance of poly lactic-co-glycolic acid (PLGA)-based nanoplatforms (PLGA NPFs) in combating microbial biofilm. Further research is required to assess how PLGA Nanoparticles (NPs) inhibit biofilm formation and eradicate established biofilms. The review article titled “Cinnamomum: The New Therapeutic Agents for Inhibition of Bacterial and Fungal Biofilm-Associated Infection” from Didehdar et al. enlightens the promising usage of Cinnamomum in the prevention of bacterial and fungal biofilms and their potential usage in a coating of medical materials to suppress biofilm formation. Exceptionally, Cinnamaldehyde has shown potential anti-biofilm effects against different microorganisms. It can be used as a substitute for antibiotics in treating infections related to biofilm. Further research is needed to confirm the molecular interactions between Cinnamomum and the cellular pathways of microorganisms, despite some studies suggesting such interactions. The study titled “Strategies to prevent, curb and eliminate biofilm formation based on the characteristics of various periods in one biofilm life cycle” by Ma et al. puts forward the ambiguity in the research process for the prevention of the formation of bacterial biofilm which elaborates multifactor approach and need of a tailor-made scientific approach for the innovation to curb the issue of formation of bacterial biofilm. In a nutshell, the Research Topic provides a summary of recent challenges and potential mitigation strategies for the prevention of the formation of bacterial biofilm, which is an unsolved and persistent issue that poses a serious threat to human survival. Additionally, it provides an opportunity for the scientific community working in this field to address the issues on a large scale in future research along with the potential benefits of utilizing nanotechnology as a novel formulation technology to address the growing problem of the formation of bacterial biofilm

## Author contributions

All authors listed have made a substantial, direct, and intellectual contribution to the work and approved it for publication.
